# Coronary Artery Aneurysms: Analysis of Comorbidities from the National Inpatient Sample

**DOI:** 10.7759/cureus.4876

**Published:** 2019-06-10

**Authors:** Varun Tandon, Aysha A Tandon, Manish Kumar, Christian M Mosebach, Kathir Balakumaran

**Affiliations:** 1 Internal Medicine, University of Arizona College of Medicine - Phoenix, Phoenix, USA; 2 Internal Medicine, University of Connecticut Health Center, Farmington, USA; 3 Cardiology, University of Connecticut Health Center, Farmington, USA

**Keywords:** coronary artery aneurysm, coronary arteries, coronary artery ectasia

## Abstract

Introduction

Coronary artery aneurysms (CAA) are not commonly seen in the general population, with an incidence of approximately 0.37% to 2.53%. Patients are typically asymptomatic but symptomatic presentation varies from dyspnea and angina to myocardial infarction or even sudden cardiac death.

Methods

We conducted a retrospective analysis using the National Inpatient Sample Healthcare Cost and Utilization Project (NIS-HCUP) database to query individuals with the diagnosis of CAA with the International Classification of Disease (ICD) code 414.11 in all discharge diagnoses for the years 2006-2014. History of Kawasaki disease was determined by ICD code 446.1.

Results

From 2006 to 2014, there were 23,033 patients identified with CAA, correlating to approximately one case per 10,000 patients or an incidence of close to 0.01%. Of this, 1,405 or approximately 6.1% of these patients had Kawasaki disease. The mortality rate of CAA was 1.79%. In terms of demographics, Caucasians were the most likely to develop CAA, with 73.8% of cases. The mean age was 61.2 years, with a mean length of stay of 5.1 days. The average cost of admission was $70892. The presence of perivascular disease (15.5% vs 4.5% p<0.05), hypertension (66.1% vs 39.1% p<0.05), chronic lung disease (20.2% vs 15.1% p<0.05), diabetes (21.7% vs 15% p<0.05), renal failure (11% vs 8.8% p<0.05), coagulopathy (6.2% vs 3.4% p<0.05), and obesity (13.1% vs 8.2% p<0.05) were all risk factors for CAA as compared to those without. It was noted that weight loss (3.28% vs 1.91% p<0.05), electrolyte abnormalities (18.2% vs 15.5% p<0.05), and blood loss (2.1% vs 0.9% p<0.05) were protective of CAA.

Conclusion

CAA risk factors appear to be similar to those of coronary artery disease risk factors, with hypertension, diabetes, perivascular disease, and renal failure. Additionally, obesity was noted to be a risk factor but weight loss appeared to be protective. Interestingly, Kawasaki disease was seen at almost similar rates as these cardiac risk factors. The incidence of CAA we found, of almost 0.01%, is much less than in the quoted literature, however, previous studies did not have as many cases as our study.

## Introduction

Coronary artery aneurysms (CAA) or coronary artery ectasias are not common, with a quoted incidence of 0.37%-2.53% [1]. Clinically, a CAA is defined as an aneurysmal dilation of the coronary artery of 1.5 times the diameter of the adjacent normal coronary artery based on the coronary artery surgery study (CASS) registry [2]. Most patients are asymptomatic but symptomatic presentation may include dyspnea, angina, fistula formation, myocardial infarction, or even sudden cardiac death [1]. Fear arises upon its discovery for the potential rupture of the aneurysm with its entailing catastrophic results. Previous studies and reports describe the risk factors of hypertension, known coronary artery disease, as well as Kawasaki disease leading to CAA, however, there has not been wide-spread data on the other comorbidities associated with CAA [3]. We propose a study to evaluate the different comorbidities associated with CAA to better identify those at risk.

## Materials and methods

Data source

Data were queried via the National Inpatient Sample (NIS) developed in part by the Healthcare Cost and Utilization Project (HCUP). The NIS database is one of the largest publicly available all-payer databases in the United States. The dataset per year represents approximately 35 million hospital admissions nationally.

Study population

We queried the NIS database from the year 2006 to 2014 to look at individuals with the diagnosis of CAA with the International Classification of Disease (ICD) code of 414.11 in all discharge diagnoses for the years 2006-2014.

Variables

Patients diagnosed with CAA were then analyzed to look at demographics. This included in-hospital mortality, age, and gender. Total hospital charges and length of stay were also analyzed. We additionally identified the average income quartile calculated from the patient's residential zip code as well as the number of procedures performed during the hospitalization. In-hospital mortality scores were based on the Elixhauser Comorbidity Index, which is a dichotomous method of categorizing the comorbidities of patients based on ICD codes. Comorbidities were established using the Elixhauser-determined comorbidities. History of Kawasaki disease was determined by ICD code 446.1.

Statistical analysis

Statistical analyses were performed using SAS software, version 9.4 (SAS Institute, North Carolina, US). Data were weighted as provided by HCUP in order to generate national estimates. Categorical variables were analyzed using chi-square analysis and continuous variables were tested using analysis of variance (ANOVA). For further delineation of the analysis, multivariate regression was also used to determine inter-racial discrepancies and disparities. The level of statistical significance (α) was chosen as 5%.

## Results

Through the timeframe of our study from 2006-2014, 333,933,821 hospital admissions were analyzed. During this timeframe, there were 23,033 patients who had CAA, correlating to almost one case per 10,000 patients. Of this amount, 1,405 patients or approximately 6.1% of patients were found to also have Kawasaki disease. In terms of demographics, females represented 29.98% of patients (6,900). In addition, Caucasians were found to be the most frequent race involved, with a total of 14,336 patients comprising 73.8% of total CAA patients. During the study period, 412 patients died with CAA during their hospitalization, correlating to a mortality rate of approximately 1.79%. The average age of patients with CAA was 61.2 years, with the average length of stay during the hospitalization being 5.12 days. The average total cost billed for the hospitalization for patients with CAA was roughly $70,892, with the average income quartile indicated by the patient’s zip code as 2.47. The number of procedures on average during a CAA patient’s stay was 4.68. The determination of the Elixhauser mortality index score was a mean of 2.94 with a readmission index score of 9.65.

When comparing the presence of the various Elixhauser comorbidities between the general population as well as with patients with CAA, there was no statistical difference in the presence of having hypothyroidism, peptic ulcer disease, lymphoma, or deficiency anemias (Table 1). The presence of perivascular disease (15.5% vs 4.5% p<0.05), hypertension (66.1% vs 39.1% p<0.05), chronic lung disease (20.2% vs 15.1% p<0.05), diabetes (21.7% vs 15% p<0.05), renal failure (11% vs 8.8% p<0.05), coagulopathy (6.2% vs 3.4% p<0.05), and obesity (13.1% vs 8.2% p<0.05) were all risk factors for CAA as compared to those without. Interestingly, having diabetes with chronic complications seemed to be a protective risk factor (2.8% vs 3.5% p<0.05). Additionally, it was noted that the presence of aquired immunodeficiency syndrome (AIDS) (0.20% vs 0.07% p<0.05), valvular heart disease (2.86% vs 2.23% p<0.05), pulmonary circulation disorders (1.48% vs 0.79% p<0.05), paralysis (1.99% vs 1.09% p<0.05), other neurological disorders (5.98% vs 3.90% p<0.05), liver disease (2.18% vs 1.12% p<0.05), metastatic cancer (1.75% vs 0.64% p<0.05), solid tumor without metastasis (1.61% vs 1.11% p<0.05), weight loss (3.28% vs 1.91% p<0.05), electrolyte abnormalities (18.2% vs 15.5% p<0.05), blood loss (2.1% vs 0.9% p<0.05), alcohol abuse (3.67% vs 2.11% p<0.05), drug abuse (3.40% vs 1.45% p<0.05), psychoses (3.56% vs 2.13% p<0.05), and depression (8.25% vs 6.24% p<0.05) were seen less in CAA patients and thus more likely to be a protective factor.

**Table 1:** Comparison of various Elixhauser comorbidities Table representing the various Elixhauser comorbidities amongst those with CAA and those without in the general population from the years 2006-2014. P-values were determined via Chi-square test analysis. CAA: Coronary Artery Aneurysm; Non-CAA: Without Coronary Artery Aneurysm; AIDS: Acquired Immunodeficiency Syndrome

Comorbid	Non-CAA Number	Non-CAA %	CAA Number	CAA %	p-value
AIDS	665961	0.20	15	0.07	0.0417
Valvular Heart Disease	9543390	2.86	514	2.23	0.0096
Pulmonary Circulation Disorders	4933582	1.48	183	0.79	0.0001
Peripheral Vascular Disorders	14917583	4.47	3590	15.59	0.0001
Hypertension	130596290	39.11	15230	66.12	0.0001
Paralysis	6629570	1.99	251	1.09	0.0001
Other Neurological Disorders	19951776	5.98	898	3.90	0.0001
Chronic Lung Disease	50266538	15.05	4660	20.23	0.0001
Diabetes Mellitus (Uncomplicated)	50136215	15.01	4999	21.70	0.0001
Diabetes Mellitus (With Chronic Complications)	11588514	3.47	646	2.81	0.0127
Hypothyroidism	28747905	8.61	1943	8.44	0.6685
Renal Failure	29232887	8.75	2543	11.04	0.0001
Liver Disease	7271465	2.18	259	1.12	0.0001
Peptic Ulcer Disease	93899	0.03	14	0.06	0.1572
Lymphoma	2049635	0.61	110	0.48	0.2233
Metastatic Cancer	5847834	1.75	148	0.64	0.0001
Solid Tumor without Metastasis	5392484	1.61	255	1.11	0.0055
Rheumatoid Arthritis/Collagen Vascular Disease	6832140	2.05	477	2.07	0.9104
Coagulopathy	11424965	3.42	1432	6.22	0.0001
Obesity	27499411	8.24	3023	13.12	0.0001
Weight Loss	10959048	3.28	439	1.91	0.0001
Fluid and Electrolyte Disorders	60630152	18.16	3577	15.53	0.0001
Chronic Blood Loss Anemia	7090242	2.12	226	0.98	0.0001
Deficiency Anemias	44457624	13.31	2901	12.59	0.1447
Alcohol Abuse	12255003	3.67	486	2.11	0.0001
Drug Abuse	11360965	3.40	333	1.45	0.0001
Psychoses	11881444	3.56	491	2.13	0.0001
Depression	27546832	8.25	1438	6.24	0.0001

## Discussion

Atherosclerosis has been a well-established risk factor for CAA for decades owing to the histologic features of lipid deposition as well as calcification and fibrosis [3-4]. Other risk factors or comorbidities have not been frequently well-studied. Our study attempted to gain perspective into what these other risk factors may include. As early as 1978, it was believed that gender was a risk factor, with males being more predicted to develop CAA [5]. Our study corroborates this as we observed that of all CAA patients, approximately 70% were male. A large majority of the patients were found to be Caucasian (73.8%). We additionally found that risk factors for coronary artery disease also tended to be risk factors for CAA. These included the presence of peripheral vascular disease, hypertension, diabetes mellitus without complication, renal failure, and obesity. Of the comorbidities that were analyzed, hypertension seemed to be the most associated risk factor for CAA with a prevalence of 66.1% followed by diabetes mellitus without a complication at 21.7%. Fascinatingly, previous literature had found that patients with diabetes mellitus had a lower incidence of CAA, which was believed to be due to the downregulation of matrix metalloproteinases promoting negative remodeling in response to atherosclerosis [2,6-7]. This likely explains the fact that we determined that patients with diabetes mellitus with complications seemed to have a possible protective effect against CAA. Obesity, especially when combined with metabolic syndrome, is a known risk factor for coronary artery disease. It also appeared to be a risk factor for CAA, but vice versa, weight loss was associated with a lower prevalence of CAA.

We also noted that the presence of chronic lung disease was associated with a higher risk of CAA. Interestingly, pulmonary circulation disorders were noted to be less prevalent in those with CAA as compared to their counterparts. The Elixhauser coding for pulmonary circulation disorders does include cor-pulmonale, which is the end-stage for chronic lung disease, however, it does also include pulmonary embolism [8]. Given that pulmonary embolism is incorporated into this coding category, this may be the cause that it appears as though pulmonary circulation disorders are protective for CAA. Interestingly, rheumatoid arthritis/collagen vascular disease was not associated with a statistically significant difference in prevalence between patients with CAA and those without. This is especially peculiar, as determined above, and previous studies that coronary artery disease and its risk factor increases the likelihood and development of CAA. Rheumatoid arthritis is known to be a risk factor for the development of coronary artery disease and the lack of association with CAA may reflect an alternative mechanism of atherosclerosis in coronary artery disease [9-10]. Additionally, typical collagen vascular disease, such as Ehlers-Danlos and Marfan syndrome, are not amongst the coding for collagen vascular disease, although other ones, including polyarteritis nodosa, systemic lupus erythematosus, and Behcet disease, are incorporated [4,8,11].

Although we found that drug abuse history appeared to be more protective of CAA, it is noted in the literature that cocaine is a possible risk factor and, therefore, it is difficult to conclude this comorbidity to truly be protective [11-12]. Additionally, sickle cell disease has been noted to be a risk factor in the literature, however, it is not part of the Elixhauser comorbidities [7].

It has been documented that if untreated, Kawasaki disease may progress to CAA in up to 25% of patients [13-14]. Remarkably, Kawasaki disease in our study was seen the least out of all the comorbidities associated with CAA, at a prevalence rate of almost one-quarter of that described in the literature.

Although it is cited that the five-year mortality rate of patients with CAA is 71%, we found the mortality rate during this time frame to be only 1.79%, albeit during only one hospitalization [15]. Lastly, the overall incidence of CAA was less than that described in the literature at 0.1% as compared to 0.37%-2.53% [1].

## Conclusions

CAA risk factors include those similar to coronary artery disease with hypertension, diabetes mellitus, perivascular disease, and renal failure. Also, chronic lung disease and obesity were seen as risk factors while weight loss, electrolyte abnormalities, as well as a history of blood loss were noted to be protective of CAA. We found a lower incidence of CAA than that typically described in the literature. Additionally, the rate at which we found Kawasaki to be associated with CAA was much less than that documented previously.

Our study has several limitations. The first is that we used retrospective data using billable codes. Being that coding may not be exact for each patient, it is possible that this may have influenced our conclusion about associated comorbidities with CAA. The main limitation of the Elixhauser Comorbidity Index score is that it is based on ICD-9 codes, which do not distinguish between complications occurring during a hospitalization vs a chronic comorbid condition. In conclusion, our study sheds light on the additional comorbidities associated with CAA that are not typically known as well as those that may be noted to be protective.
